# The Impact of Probiotic Supplementation on Cognitive, Pathological and Metabolic Markers in a Transgenic Mouse Model of Alzheimer’s Disease

**DOI:** 10.3389/fnins.2022.843105

**Published:** 2022-05-24

**Authors:** Thomas S. Webberley, Giulia Masetti, Ryan J. Bevan, Joshua Kerry-Smith, Alison A. Jack, Daryn R. Michael, Sophie Thomas, Maria Glymenaki, Jia Li, Julie A. K. McDonald, Daniel John, James E. Morgan, Julian R. Marchesi, Mark A. Good, Sue F. Plummer, Timothy R. Hughes

**Affiliations:** ^1^Division of Infection and Immunity, School of Medicine, Cardiff University, Cardiff, United Kingdom; ^2^Cultech Ltd., Port Talbot, United Kingdom; ^3^UK Dementia Research Institute, Cardiff University, Cardiff, United Kingdom; ^4^School of Optometry and Vision Sciences, Cardiff University, Cardiff, United Kingdom; ^5^Division of Digestive Diseases, Department of Metabolism, Digestion and Reproduction, Faculty of Medicine, Imperial College London, London, United Kingdom; ^6^Department of Life Sciences, Faculty of Natural Sciences, Imperial College London, London, United Kingdom; ^7^School of Psychology, Cardiff University, Cardiff, United Kingdom

**Keywords:** Alzheimer’s disease, neurodegeneration, probiotics, microbiota, obesity, inflammation

## Abstract

Brain degenerative disorders such as Alzheimer’s disease (AD) can be exacerbated by aberrant metabolism. Supplementation with probiotic bacteria is emerging as a promising preventative strategy for both neurodegeneration and metabolic syndrome. In this study, we assess the impact of the Lab4b probiotic consortium on (i) cognitive and pathological markers of AD progression and (ii) metabolic status in 3xTg-AD mice subjected to metabolic challenge with a high fat diet. The group receiving the probiotic performed better in the novel object recognition test and displayed higher hippocampal neuronal spine density than the control group at the end of the 12 weeks intervention period. These changes were accompanied by differences in localised (brain) and systemic anti-inflammatory responses that favoured the Probiotic group together with the prevention of diet induced weight gain and hypercholesterolaemia and the modulation of liver function. Compositional differences between the faecal microbiotas of the study groups included a lower Firmicutes:Bacteroidetes ratio and less numbers of viable yeast in the Probiotic group compared to the Control. The results illustrate the potential of the Lab4b probiotic as a neuroprotective agent and encourage further studies with human participants.

## Introduction

The gut microbiota is a complex community of microorganisms heavily influenced by host genetics, diet and the environment and has a major impact upon host physiology (Fan and Pedersen, 2020). A bidirectional communication pathway exists between the gut microbiota and the central nervous system (CNS), termed the “gut-microbiota-brain axis,” and there is increasing evidence linking disruptions of this pathway with neurological disease development (Morais et al., 2020). It is emerging that neurodegenerative conditions such as Parkinson’s disease (PD) and Alzheimer’s disease (AD) are associated with perturbations to the gut microbiota (Zhuang et al., 2018; Zhang et al., 2020).

AD is the most common form of dementia with estimates of 850,000 sufferers in the United Kingdom alone (Alzheimer’s Society’s, 2019) and over 50 million worldwide (World Health Organisation [WHO], 2020). AD is characterised by progressive loss of cognitive function and is thought to be driven, at least in part, by inflammatory damage and the deposition of amyloid beta (Aβ) plaques in the cortex and hippocampal regions of the brain leading to progressive synaptic loss and neuronal death (John and Reddy, 2020). Ageing is the strongest risk factor for AD and there is currently no cure for established disease, only strategies to slow progression and alleviate symptom severity (Yiannopoulou and Papageorgiou, 2020). Other risk factors for AD include the impact of metabolic syndrome and excess body weight during early/mid-life that has been shown to double the risk of developing AD during later life (Anstey et al., 2011). Numerous studies have shown that diet-induced obesity in genetically modified amyloid precursor protein mice leads to accelerated disease progression (Julien et al., 2010; Knight et al., 2014; Kim et al., 2017) and with the increasing prevalence of obesity in society (World Health Organisation [WHO], 2018) there is a need for strategies to reduce excessive body weight gain as a means of reducing the risk of AD.

Probiotics are becoming recognised for their ability to impart immunological and metabolic benefits (including weight loss) on the host (Tenorio-Jimenez et al., 2020) and there is growing support for the use of probiotic supplementation to attenuate/prevent AD pathology in AD mice (Bonfili et al., 2017, 2018, 2020; Tan et al., 2022) and humans (Zhu et al., 2021). The Lab4b consortium, which comprises *Lactobacillus paracasei*, *Lactobacillus salivarius*, together with two strains of bifidobacteria, can enhance the immune responses in macrophages (Davies et al., 2018) and illicit protective effects in response to neuronal oxidative stress (Michael et al., 2019). In the current study, we tested the hypothesis that the Lab4b probiotic would support cognitive function, ameliorate neurodegeneration, and improve metabolic status in metabolically challenged 3xTg-AD prone mice.

## Materials and Methods

### Reagents

All reagents were purchased from Sigma-Aldrich/Merck (Poole, United Kingdom) unless otherwise stated.

### Ethics

Mouse experiments were performed under the United Kingdom Home Office Project Licence (PPL 30/3220). The 3xTg-AD mice were produced from an in-house colony, founders originally purchased from JAX laboratories (Bar Harbor, ME, United States, strain 004807) after generation by Frank LaFerla (University of California, Irvine).

### Mouse Husbandry and Study Design

Twenty 3xTg-AD male mice were fed standard chow diets, *ad libitum* (Teklad rodent diet, Envigo) until 12 weeks of age before they were randomly assigned to two groups (10/group); one group received high fat diet alone for 12 weeks (termed the “Control” group) and the second group received HFD supplemented with the Lab4b probiotic providing a minimum of 5 × 10^8^ colony forming unit (cfu)/mouse/day for 12 weeks (termed the “Probiotic” group). Mice were housed in scantainer vented cages (2–4 mice per cage) in a light and temperature-controlled environment (12 h on/off light at 22°C) and had *ad libitum* access to all diet and water. The HFD was provided to promote AD pathologies in these mice (Julien et al., 2010; Knight et al., 2014; Kim et al., 2017) and comprised 21% (w/w) pork lard with 0.15% (w/w) cholesterol (Special Diets Services, Witham, United Kingdom; product code: 821424). Full dietary composition of both the chow and HFD are shown in Supplementary Tables 1, 2. Typically, male 3xTg mice display cognitive deficits from 2 months of age (Billings et al., 2005) and amyloid deposition in the brain from 6 months of age (Oddo et al., 2003). The Lab4b probiotic was administered by mixing with feed and comprised *Lactobacillus salivarius* CUL61 (NCIMB 30211), *Lactobacillus paracasei* CUL08 (NCIMB 30154), *Bifidobacterium bifidum* CUL20 (NCIMB 30153), and *Bifidobacterium animalis* subsp. *lactis* CUL34 (NCIMB 30172). Mice were weighed every 2 weeks over the duration of the intervention period. Mice were routinely screened for health over the duration of the study and no indications of infection or poor health were observed.

### Sample Collection

At the end of the study, faecal samples were collected and stored in anaerobic bags (Genbag, bioMérieux) at −80°C. The mice were euthanised by CO_2_ inhalation and whole blood was obtained by cardiac exsanguination into heparinised tubes, centrifugation at 3,000 × *g* for 5 min and collection of plasma. The brain was removed by dissection and the right hemisphere was fixed in 4% paraformaldehyde (for histology) or analysed immediately for hippocampal slices (DiOlistic neuronal labelling). The left hemisphere of the brain was extracted and snap-frozen in liquid nitrogen (for RNA isolation and gene expression analyses). Whole livers were extracted, snap-frozen in liquid nitrogen and stored at −80°C (for RNA isolation, gene expression, and metabolite analyses).

### Behavioural Testing

All behavioural testing was performed in a custom-made plastic test arena [39 cm (height) × 39 cm (width) × 39 cm (length)] placed in a class II laminar flow hood. The base of the arena was covered in a thin layer of wood chip sawdust. Novel object recognition (NOR) testing was performed in three stages spanning 2 days. On day 1, each mouse was allowed to explore the empty test arena for 10 min (habituation phase) before being returned to its normal housing. The next day, the mouse was placed in an empty arena for 10 min (open field test) before returning to its normal housing for 30 min. It was then placed back in the test arena along with two identical objects for 10 min (training phase) before being returned to its normal housing for 30 min followed by a further 10 min in the test arena containing a novel object in place of one of the identical objects (test phase). Results were expressed as the discrimination ratio (DR) calculated by dividing the time spent exploring the novel object (head oriented toward and within 2 cm) by the time spent exploring the familiar object plus the novel object. A reduction in DR denotes decreased preference and perception of a novel object and therefore an indicator of impaired memory. Mouse movements were recorded using a GoPro HERO session camera (GoPro, United States) positioned directly above the test arena and movements analysed using EthoVision XT 13 software (Noldus, Netherlands). The familiar and novel objects were similar in size but differed in colour and shape. Objects were rubbed with the cage sawdust to mask any potential scent from them and encourage the test to be more driven by visual awareness. The experimenter was blinded to the group allocation during the test.

### Hippocampal Dendritic Spines

Hippocampal slices (right brain hemisphere) were subjected to DiOlistic gene gun labelling to assess the density of dendritic spines on CA1 apical dendrites according to a previous described method (Gan et al., 2000; Binley et al., 2016). *Bullet preparation:* Briefly, the fluorometric dyes DiI (Life Technologies, United States) were dissolved in dichloromethane to coat tungsten particles (Bio-Rad, United States). DiI coated tungsten particles were funnelled into ethylene tetrafluoroethylene (ETFE) tubing for ballistic DiOlistic labelling of the hippocampal slices (200 μm thick). Slices were subjected to 100 psi particle delivery using a Helios Gene Gun (Bio-Rad, United States) through a 3.0 μm pore size cell culture insert. Prevention of over labelling and aggregated particles was minimised by frequent replacement of inserts. Hippocampal slices were placed in Neurobasal-A media (Life Technologies, United States) and incubated for 20 min at 37°C with 5% CO_2_ to facilitate dye diffusion. Slices were fixed in 4% paraformaldehyde (PFA) for 30 min at room temperature, nuclear stained with Hoechst 33342 and mounted in FluorSave. *Spine imaging*: DiI labelled CA1 neurones were imaged for their dendritic spines on apical dendrites within the striatum radium region using the Leica SP8 laser-scanning confocal microscope with lightning deconvolution (Leica Microsystems, Germany). Dendritic segments longer than 30 μm were 3D reconstructed using the Filament Tracer module in Bitplane Imaris software v9.3.1 and spines subtyped with the Spine Classifier MATLAB extension. Spines were morphologically classified based on predetermined settings as follows: spines <0.8 μm: stubby, >0.8 but <3 μm and with a spine head diameter greater than neck width: mushroom spines; other spines >0.8 but <3 μm: thin spines. Each reconstructed dendrite segment was manually checked to ensure correct spine detections. Each type of spine was averaged from at least three separate values from each mouse.

### Brain Histology

The right brain hemisphere was fixed in 4% (w/v) PFA for ≥24 h before immersion in 30% (w/v) sucrose solution for up to 72 h. The brains were then placed in moulds with Optimal Cutting Temperature (OCT) Embedding Matrix (CellPath, United Kingdom) and frozen in dry ice and stored at −80°C prior to sectioning. Sections were cut at on a sagittal plane using a Cryostat (10 μm), mounted onto slides (X-tra Slides, Leica Biosystems) and stored at −80°C prior to staining.

For staining, sections were briefly exposed to citrate-based heat-induced antigen retrieval before immersion in BLOXALL solution (SP-6000, Vector Laboratories, Oxford, United Kingdom) for 10 min at room temperature (RT) followed by 30 min in 2.5% (v/v) normal horse serum at room temperature (S-2000, Vector Laboratories, Oxford, United Kingdom). Primary ionised calcium-binding adaptor protein-1 (Iba-1) antibody (Wako, Japan) was diluted 1:1000 in 2.5% (v/v) horse serum in PBS and incubated overnight at 4°C. Slides were stained with secondary species-specific biotinylated antibodies (Vector Laboratories, Oxford, United Kingdom) followed by the VECTASTAIN Elite ABC-HRP Kit (PK-6100, Vector Laboratories, Oxford, United Kingdom), and finally, ImmPACT Diaminobenzidine (DAB) Peroxidase (HRP) Substrate (Vector Laboratories, Oxford, United Kingdom) in accordance with the manufacturer’s instructions. Sections were then counterstained using haematoxylin, dehydrated in graded alcohols and cleared in xylene followed by DPX mounting under coverslips. Slides were scanned using the Axio Scan.Z1 Digital Slide Scanner (Carl Zeiss Ltd., United Kingdom) and analysis of the cortex and hippocampus was performed using QuPath (Bankhead et al., 2017). Analysis was performed on one brain section per mouse.

For detecting and quantifying the density of amyloid plaques, cryosections were stained with Thioflavin S solution. Briefly, slides were air-dried and stained with filtered (0.2 μm) 0.1% Thioflavin S solution in 70% (v/v) ethanol for 10 min. After brief washes in 70% (v/v) ethanol, 50% (v/v) ethanol and water, the slides were cover slipped in FluorSave. Plaques coverage in the hippocampus and cortex were imaged using a Leica SP8 confocal microscope (Leica Microsystems, United Kingdom) and analysed in Bitplane Imaris software v9.3.1 using the volume function.

### Tissue Extraction of RNA

Brain (left hemisphere) and liver tissues were homogenised with RiboZol (VWR, United States) for 3 × 20 s in a Fast Prep-24 Bead Beater (MPBIO, United States) with cooling on ice between each round. The homogenised tissues were extracted with chloroform and centrifuged at 12,000 *g* for 15 min at 4°C. The aqueous phase was extracted with isopropanol and centrifuged at 12,000 *g* for 10 min at 4°C. The RNA pellets were washed three times with ethanol (75% v/v), resuspended in RNAse free double-distilled (ddH_2_O) and quantified by Qubit (Thermo Fisher Scientific, United States).

### Reverse Transcriptase Quantitative Polymerase Chain Reaction of Whole Tissue Extracts

cDNA was prepared from the RNA using a High-Capacity cDNA Reverse Transcription Kit (Applied Biosystems, United States) in accordance with the manufacturer’s instructions. cDNA (10 ng) was added to each qPCR reaction comprising iTaq™ Universal SYBR^®^ Green Supermix (Applied Biosystems, United States) and 50 nM of each gene-specific primer (Supplementary Table 3). PCR was carried out on a CFX96 (Bio-Rad, United States) using the following cycles parameters: Initial melting (95°C for 5 min) followed by 40 cycles of melting (94°C for 15 s), annealing (60°C for 15 s), and extension (72°C for 30 s). Relative transcript levels were determined using 2-(^ΔC*t*1–ΔC^*^t^*^2^) where ΔC*t* represents the difference between the threshold cycle (C*t*) for the target gene and β-actin transcript levels and are expressed as a ratio of the Control group.

### Plasma Lipid and Cytokine Profiling

Plasma levels of total cholesterol (TC), high-density lipoprotein (HDL) and low-density lipoprotein (LDL)/very low-density lipoprotein (vLDL) were assayed using the Cholesterol assay kit (Abcam, United Kingdom) according to manufacturer’s instructions. Plasma levels of triglycerides (TG) were assayed using the Triglyceride assay kit (Abcam) according to manufacturer’s instructions. The following cytokines: Interferon (IFN)-γ, interleukin (IL)-2, IL-4, IL-5, IL-6, IL-10, IL-1β, Keratinocyte chemoattractant/growth regulated oncogene (KC/GRO), tumour necrosis factor (TNF)-α were measured in plasma [MSD multiplex platform performed by Central Biotechnology Services (CBS), Cardiff].

### ^1^H NMR Spectroscopic Analysis of Tissue and Data Analysis

Samples preparation is described in detail in the Supplementary Methods. Sample analysis was performed as previously described (Glymenaki et al., 2017) using a Bruker DRX 600 MHz spectrometer (Bruker, Rheinstetten, Germany).

#### Microbiome Analysis

##### DNA Extraction From Faeces and 16S rRNA Gene Sequencing

DNA was extracted from faeces using the QIAamp^®^ Fast DNA Stool Mini Kit (Qiagen, Germany) according to the manufacturer’s instructions. Briefly, samples were homogenised in Matrix Lysing D tubes (MPBIO) and a FastPrep^®^-24 bead beater (MPBIO, United States). Eluted genomic DNA were quantified using a Qubit^®^ (Thermo Fisher Scientific, United States) and normalised to 5 ng in tris-acetate-ethylenediaminetetraacetic acid (TAE) and stored at −20°C. Sample libraries were prepared following Illumina’s 16S Metagenomic Sequencing Library Preparation Protocol with the following modifications. First, V1–V2 regions of the 16S rRNA gene were amplified in addition to bifidobacteria sequences (Moshkelgosha et al., 2018). The index PCR were cleaned-up and normalised using the SequalPrep Normalization Plate Kit (Life Technologies, Paisley, United Kingdom). Sample libraries were quantified using the NEBNext Library Quant Kit for Illumina (New England Biolabs, Hitchin, United Kingdom). Sequencing was performed on an Illumina MiSeq platform (Illumina Inc., Saffron Walden, United Kingdom) using the MiSeq Reagent Kit v3 (Illumina) and paired-end 300 base pairs (bp) chemistry. Negative controls for the sequencing were reactions containing no template DNA. The positive control comprised the defined bacterial community MCM1 (composition in Supplementary Table 4). Paired-end 16S rRNA gene sequencing reads were trimmed, chimaera reduced and paired ends were merged using DADA2 (Callahan et al., 2016) to obtain sequence variants which were aligned against the SILVA database to assign taxonomy (Quast et al., 2013).

##### Analysis of the Bacterial Composition in Faeces Using Next Generation Sequencing Data

Alpha (Chao1 and Shannon) and beta diversity measures were obtained in the R package “phyloseq” (McMurdie and Holmes, 2013). Weighted UniFrac matrix (with 10,000 permutations) (Lozupone and Knight, 2005) were calculated using the Adonis function of the R Vegan package (Anderson, 2001).

##### *Ex vivo* Culture of Viable Micro-Organisms in Faeces

Numbers of viable lactobacilli, enterobacteria, enterococci, coliforms, staphylococci, yeast, bifidobacteria, bacteroides, clostridia, total aerobes and total anaerobes were enumerated on selective media in accordance with a previously described method (Baker et al., 2021).

### Statistical Analysis

All statistics were performed using GraphPad PRISM (version 8.2.1, CA, United States) or R package (Version 3.1.3) unless otherwise stated with *p* < 0.05 considered statistically significant.

Prior to statistical analysis, the data were tested for normality using the Shapiro–Wilk test and visual inspection of Q–Q plots. For comparisons between two groups, an unpaired student’s *t*-test was performed on normally distributed (Gaussian) data and an unpaired Mann–Whitney student’s *t*-test was performed where data was not normally distributed (non-Gaussian). For multiple comparison testing on normally distributed data with equal variance between groups (determined using the Brown–Forsythe test), one-way ANOVA with Holm–Sidak *post hoc* analysis was performed. For multiple comparison testing on normally distributed data with unequal variance between groups, a Brown-Forsythe adjusted one-way ANOVA with Dunnett’s *post hoc* analysis was performed.

^1^H NMR spectral data were analysed using multivariate statistical analysis methods including principal component analysis (PCA) and orthogonal signal correction-projection to latent structures-discriminant analysis (O-PLS-DA) in MATLAB version 2017b (MathWorks, United States).

For the 16S rRNA gene sequencing data, differences in the Chao1 and Shannon indices amongst groups (Control and Probiotic group) were analysed using the non-parametric Wilcoxon rank sum test. Spatial differences of the groups were observed with a non-metric multidimensional scaling (NMDS) plot. The permutational analysis of variance (PERMANOVA) was computed on the weighted UniFrac matrix using in the Adonis function of the R Vegan package (Anderson, 2001), with cage effect as a “strata” parameter. Differential abundance of Amplicon Sequence Variant (ASV) amongst groups was analysed using DESeq2 (Love et al., 2014), and visualised with pheatmap in R.

## Results

### Lab4b Improves Cognition and Inhibits Diet Induced Weight Gain

**FIGURE 1:**
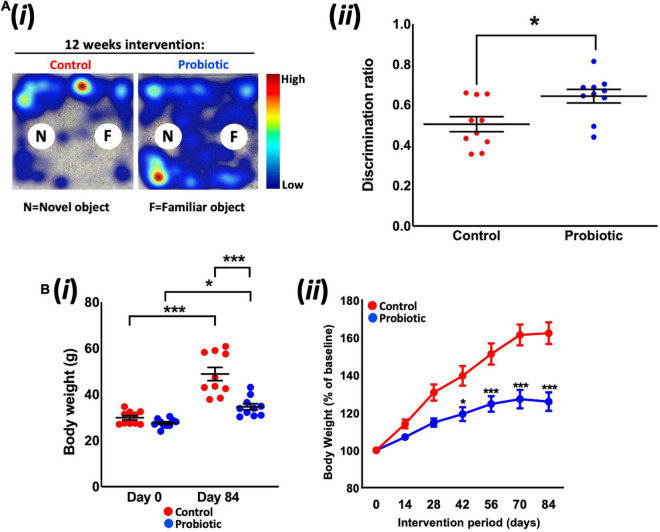
Novel object recognition and body weight. **(Ai)** Representative heat maps of mouse movements during novel object recognition (NOR) testing and **(Aii)** the discrimination ratios of the Control and Probiotic groups. **(B)** Changes in **(Bi)** absolute body weight and **(Bii)** percentage body weight over the duration of the study. Data are expressed as mean ± SEM of 10 mice per group. Values of *p* were determined using unpaired *t*-test or one-way ANOVA with Holm–Sidak *post hoc* comparisons where *p < 0.05, ***p < 0.001 or as stated.

Tracking mouse movements during the NOR task (Figure 1Ai) allowed for the calculation of a discrimination ratio (DR) that is a measure of the animals’ propensity to explore the novel object over the familiar object. After dietary intervention, the DR of the Probiotic group (0.64 ± 0.03) was significantly higher (28%, *p* = 0.0127, Figure 1Aii) than the DR of the Control group. While this analysis included all animals (10 per group) irrespective of the total amount of time spent interacting with objects, sub-group analysis was also performed on animals with more prolonged interest in objects over the 10 min period (Supplementary Figure 1). Assigning a threshold of total object interest to (i) 10 s or more, or (ii) 20 s or more (Leger et al., 2013) reduced the number of animals which could be included in the analysis, however, still yielded a significant increase in NOR in the Probiotic group compared to the Control group (10 s interest or more: *p* = 0.0067, 20 s interest or more: *p* = 0.0343, Supplementary Figure 1). Tracking mouse movements during the open field test (OFT) did not reveal any statistically significant between-group differences in distance travelled, velocity, time spent in the arena perimeter, clockwise body rotations nor time spent digging/rearing after 12 weeks supplementation (Table 1).

**TABLE 1:** Open field testing.

	After 12 weeks intervention		*p* value
	Control	Probiotic	
Total distance travelled (cm)	1509.36 ± 182.10	1570.23 ± 199.17	0.7962
Velocity (cm/s)	2.52 ± 0.30	2.62 ± 0.33	0.7962
Time spent in arena centre (s)	69.39 ± 10.43	68.65 ± 16.99	0.9706
Time spent in arena perimeter (s)	530.74 ± 10.43	531.49 ± 16.97	0.9706
Number of digging episodes (f)	31.30 ± 3.39	28.50 ± 3.56	0.5760
Number of rearings (f)	16.90 ± 1.80	14.60 ± 2.46	0.4606

*Data represent the means ± SEM from nine mice per group. Values of p were determined using an unpaired student’s t-test. Where data was not normally distributed, Mann–Whitney t-tests were performed. cm, centimetres; s, seconds; f, frequency.*

Measurement of body weight over the duration of the study revealed that the Probiotic group weighed 28.9% less than the mice in the control group after 12 weeks feeding (*p* < 0.0001, Figure 1Bi); pre-intervention weights (Day 0) were similar between groups (Control: 29.9 g ± 0.9, Probiotic: 27.6 g ± 0.6) whereas the final weights in each group were 48.8 g ± 2.9 in the control group and 34.7 g ± 1.3 in the Probiotic group (*p* < 0.0001). Both groups had gained significant amounts of weight over the duration of the study but rates of increase were lower in the Probiotic group (25.7% from Day 0 to Day 84, *p* = 0.0022, Figure 1Bii) than in the Control group (63.2% from Day 0 to Day 84, *p* = 0.0003). Statistically significant between-group differences were present after 6 weeks (42 days) supplementation and remained until the end of the study (Figure 1Bii).

### Lab4b Supplementation Minimises the Loss of Thin Neuronal Spines in the Hippocampal CA1 Region

**FIGURE 2:**
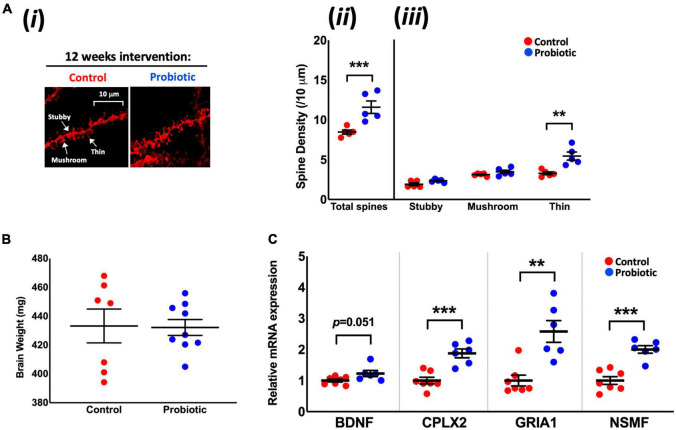
Neuronal spine density and the mRNA levels of cognition-related genes in the brain. **(Ai)** Representative DiOlistic images showing thin, stubby, and mushroom dendrites in the CA1 hippocampus region of the Control and Probiotic groups and comparisons in the density of **(Aii)** total and **(Aiii)** stubby, mushroom, and thin spines. **(B)** Average brain weights. **(C)** Relative mRNA expression levels of BDNF, CPLX2, GRIA1, and NSMF in whole brain extracts of 3xTg-AD mice. Data are expressed as mean ± SEM of five mice per group **(A)** or at least six per group **(B,C)**. Values of *p* were determined using one-way ANOVA with Holm–Sidak or using an unpaired *t*-test. **p < 0.01, ***p < 0.001 or as stated. BDNF, Brain-derived neurotrophic factor; CPLX2, Complexin-2; GRIA1, Glutamate Receptor Ionotropic AMPA Type Subunit 1; NSMF, neuronal migration factor.

DiOlistic staining of the hippocampus (Figure 2Ai) revealed significantly higher (37%) total neuronal spine density in the Probiotic group compared to the Control group (Probiotic: 11.60/10 μm ± 0.78 vs. Control: 8.47/10 μm ± 0.26, *p* < 0.0001, Figure 2Aii). At the end of the intervention, 66% more thin spines were observed in the Probiotic group compared to the Control (5.45/10 μm ± 0.50 vs. 3.29/10 μm ± 0.18, *p* = 0.0043, Figure 2Aiii) but there were no between-group differences in stubby or mushroom spines. There were no differences in brain weights between groups at the end of the study (Figure 2B). The cortex or hippocampal CA1 regions of the brain were stained by immunohistochemistry for amyloid but plaque presence could not be detected (data not shown).

The Probiotic group had significantly higher mRNA levels of complexin-2 (CPLX2, 1.88-fold, *p* = 0.0004), glutamate receptor ionotropic AMPA type subunit 1 (GRIA1, 2.57-fold, *p* = 0.0014), and neuronal migration factor (NSMF, 2.020-fold, *p* = 0.0001) compared to the Control group. There were indications of higher levels of brain-derived neurotrophic factor (BDNF, 1.22-fold, *p* = 0.0513, Figure 2C) mRNA in the Probiotic group compared to the Control. The mRNA expression levels of other neurocognitive-related genes and markers of oxidative stress and apoptosis remained unchanged between the Control and Probiotic groups (Supplementary Table 5).

### Lab4b Has No Impact Upon Immune Cell Composition in the Brain but Improves the Transcript Rates of Key Inflammatory Markers

**FIGURE 3:**
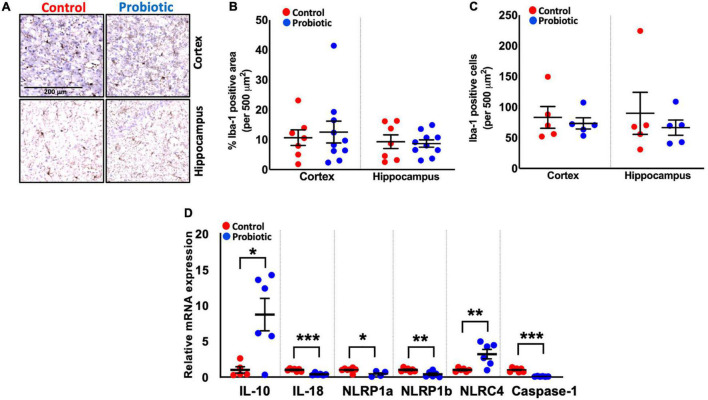
Immune cell composition and the expression levels of inflammatory genes in the brains. **(A)** Representative images showing Iba-1 staining in the cortex and hippocampus region of the Control and Probiotic groups. **(B)** Levels of Iba-1 staining and **(C)** Iba-1 positive cells in the cortex and hippocampus CA1 regions of the Control and Probiotic groups and **(D)** changes in the relative mRNA expression levels of IL-10, IL-18, NLRP1a, NLRP1b, NLRC4, and Caspase-1 in whole brain extracts of 3xTg-AD mice. Data are expressed as mean ± SEM of 10 mice per group **(A,B)** or at least five per group **(C)**. Values of *p* were determined using unpaired *t*-test. *p < 0.05, **p < 0.01, or ***p < 0.001. Iba-1, Ionised calcium binding adaptor molecule 1; IL, interleukin. NLRP1a/NLRP1b, nucleotide-binding oligomerization domain-like receptors (NLR) family pyrin domain containing 1 a/b; NLRC4, NLR family caspase activation and recruitment domains (CARD) domain-containing protein 4.

Immunohistochemical staining for Iba-1 (Figure 3A) revealed no differences in the numbers of microglial cells/macrophages in the cortex and hippocampal regions of the brain between the treatment groups at the end of the study (Figures 3B,C), however, qPCR analysis of inflammatory markers showed that the expression of interleukin (IL)-10 mRNA was higher in whole brain extracts of the Probiotic group compared to the Control group (8.94-fold, *p* = 0.014, Figure 3D). The mRNA levels of IL-18 (61%, *p* < 0.0001), NLRP1a (56%, *p* = 0.0256), NLRP1b (59%, *p* = 0.0063), and caspase-1 (91%, *p* < 0.0001) were decreased in the Probiotic group compared to the control group whereas NLRC4 expression was increased (3.25-fold, *p* = 0.0076). The mRNA expression levels of other inflammatory genes did not differ between groups and are shown in Supplementary Table 5.

### Lab4b Supplementation Alters Plasma Levels of IL-10, KC/GRO, and TNF-α

As seen in Table 1, at the end of the study, plasma levels of IL-10, KC/GRO, and TNF-α were significantly lower (53.5%, *p* = 0.0068, 43.8%, *p* = 0.0232, and 34%, *p* = 0.0167, respectively) in the Probiotic group compared to the Control group. No other significant changes were observed between groups.

### Lab4b Supplementation Improves Plasma Lipid Profile and Modulates the Expression of Lipogenic Genes in the Liver

Probiotic supplementation reduced between-group levels of vLDL/LDL by over 20% (12.04 mg/dL for the Control group vs. 9.45 mg/dL for the Probiotic group, *p* = 0.0452, Table 2) but with no effect on TC, TG, or HDL.

**TABLE 2:** Plasma cytokine/chemokine and lipid concentrations.

	After 12 weeks intervention		*p* value
	Control	Probiotic	
**Cytokine/chemokine (pg/ml)**			
Interferon-γ	0.20 ± 0.03	0.24 ± 0.03	0.3316
Interleukin-1β	1.59 ± 0.17	1.84 ± 0.36	0.5406
Interleukin-2	0.73 ± 0.13	0.72 ± 0.13	0.9332
Interleukin-5	5.12 ± 1.50	8.47 ± 2.42	0.2475
Interleukin-6	24.18 ± 3.55	34.65 ± 10.44	0.9118
Interleukin-10	117.90 ± 22.76	54.87 ± 9.53	0.0068
Keratinocyte chemoattractant/growth regulated oncogene	400.98 ± 87.76	225.42 ± 19.49	0.0232
Tumour necrosis factor-α	21.66 ± 2.30	14.29 ± 1.59	0.0167

**Plasma lipid (mg/dL)**			

Total cholesterol	113.80 ± 9.00	96.92 ± 4.93	0.2110
Very low-density lipoprotein/low density lipoprotein	12.04 ± 1.09	9.45 ± 0.51	0.0452
High-density lipoprotein	37.90 ± 3.73	32.67 ± 1.99	0.2475
Triglycerides	51.14 ± 7.80	40.42 ± 8.31	0.3598

*Data represent the means ± SEM of single measurements performed on samples from 10 mice per group. Values of p were determined using an unpaired student’s t-test. When data were not normally distributed (Shapiro–Wilk test), Mann–Whitney t-tests were performed.*

**FIGURE 4:**
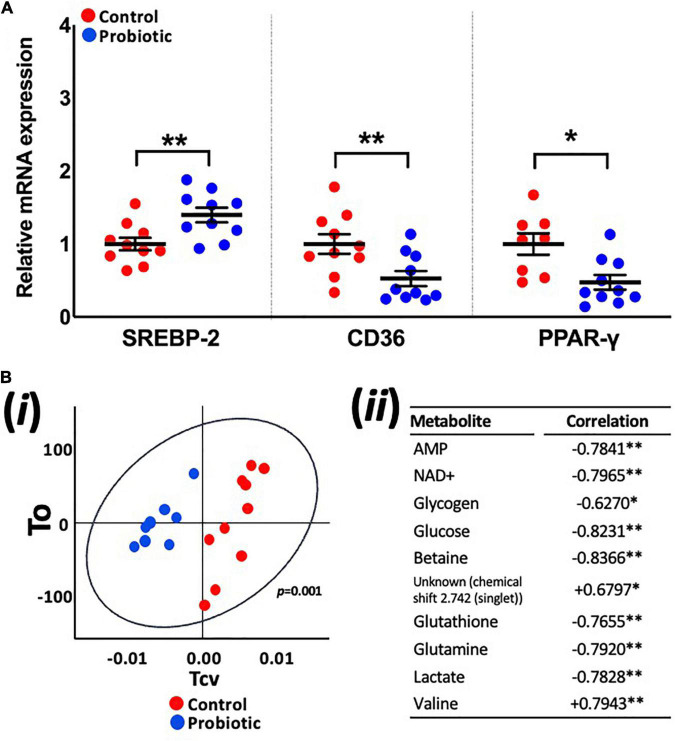
Expression levels of lipogenic genes and the metabolite profile of the liver. **(A)** The relative mRNA expression levels of SREBP-2, CD36, and PPAR-γ in the liver of 3xTg-AD mice and **(Bi)** principle component analysis (PCA) of liver metabolites in the Control and Probiotic groups and **(Bii)** correlation of individual liver metabolites in the Probiotic group (in relation to the Control group; negative values indicate higher correlation in Control group whereas positive values indicate higher correlation in the Probiotic group). Data are expressed as mean ± SEM of at least eight mice per group. Values of *p* were determined using unpaired *t*-test **(A)** or using two-tailed *t*-test **(B)**. *p < 0.05 or **p < 0.01. SREBP-2, sterol-regulatory element-binding protein-2; CD36, cluster of differentiation 36; PPAR-γ, peroxisome proliferator-activated receptor.

mRNA expression levels in the liver were higher for SREBP-2 (1.39-fold, *p* = 0.008, Figure 4A) in the Probiotic group and lower for CD36 (52%, *p* = 0.0077) and PPAR-γ (48%, *p* = 0.0118) when compared to the Control group. The expression of other lipogenic genes and markers of oxidative stress in the liver were not influenced by the probiotic (Supplementary Table 5).

### Lab4b Supplementation Influences the Metabolic Profile of the Liver

OPLS-DA analysis of metabolite profiles of liver tissues showed significant changes and were predictive of groups (Q2Yhat = 0.61, R2Yhat = 0.98, *p* = 0.001, R2X = 0.36, Figure 4Bi). Concentrations of glucose, lactate, glutathione, glutamine, glycogen, betaine, AMP, and NAD+ were higher in the Control group while valine and an unknown metabolite were higher in the Probiotic group (Figure 4Bii). Analysis of samples extracted from the brain (hippocampi, cortex, and cerebellum), faecal samples and blood plasma yielded no significant findings (Supplementary Figure 2).

### Lab4b Supplementation Impacts Upon the Composition of the Gut Microbiota

**FIGURE 5:**
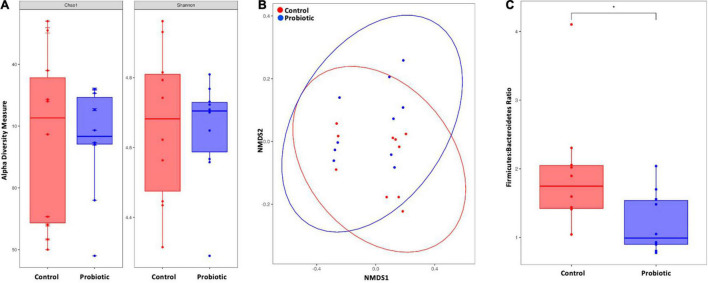
The composition of the faecal microbiota by NGS. **(A)** Alpha diversity indices of richness and diversity and **(B)** non-metric dimensional scaling (NMDS) based on weighted UniFrac distances matrix. Permutational analysis of variance was performed with caging as a strata effect. 10,000 permutations were applied. **(C)** Firmicutes-to-Bacteroidetes ratio. For **(A,C)** the Wilcox–Mann test was applied. *p < 0.05.

At the end of the study, no between-group differences in alpha diversity (Chao1 nor Shannon index) were observed (Figure 5A). Non-metric multidimensional scaling (NMDS) identified a trend toward a spatial separation between the Probiotic and Control groups at the end of the intervention period (*p* = 0.0605, 10,000 permutations; Figure 5B). The Firmicutes:Bacteroidetes ratio (F:B) was significantly lower in the Probiotic group compared to the Control group by end of the study (*p* = 0.0299, Figure 5C). No cage effects were observed during the study (data not shown).

**FIGURE 6:**
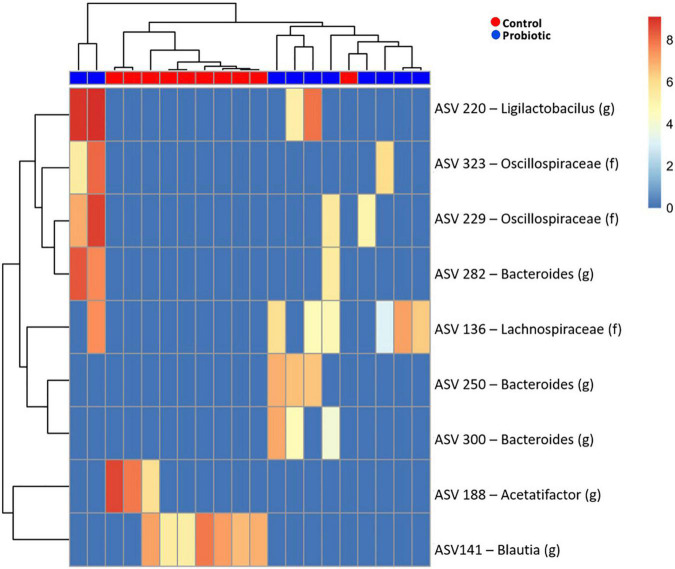
Enriched amplicon sequence variants (ASVs) in the faeces of 3xTg-AD mice. Heatmap of differential abundance of significant (*p* > 0.01) ASVs in 3xTg-AD mice when comparing the Control and Probiotic groups using DESeq2 normalisation for data after logarithmic transformation, displaying enrichment of taxa.

Amplicon sequence variant (ASV) abundance identified enrichments in both treatment groups compared to baseline (Figure 6). Blast analysis of enriched ASVs found from *Acetatifactor* and *Blautia* genera were enriched in the Control group whilst in the Probiotic group, ASVs from *Ligilactobacillus*, and *Bacteroides* genera, along with *Lachnospiraceae* and *Oscillospiraceae* family were enriched.

**FIGURE 7:**
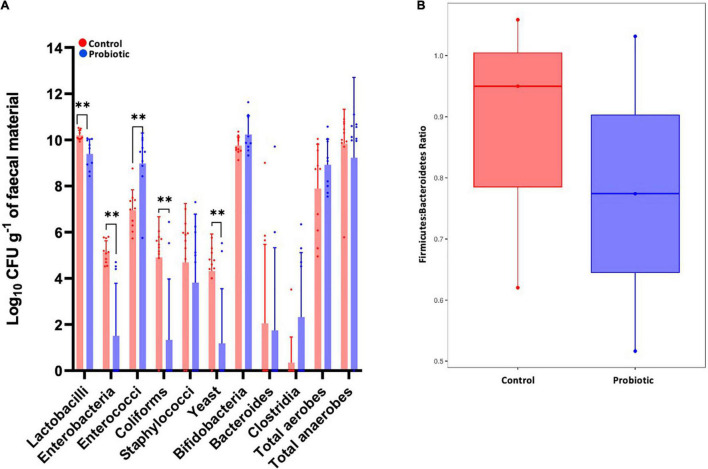
The composition of the faecal microbiota by *ex vivo* culture. Viable numbers of **(A)** common gut bacteria or **(B)** the Firmicutes-to-Bacteroidetes ratio in the faeces of 3xTg-AD mice at the end of the intervention period. For **(A)**, values of *p* were determined using an unpaired *t* test where **p < 0.01.

Between-group comparisons of the number of viable bacteria cultured from faeces identified lower numbers of lactobacilli (*p* = 0.0016, Figure 7A), enterobacteria (*p* = 0.0001), coliforms (*p* = 0.0027) and yeast (*p* = 0.0033), and higher numbers of enterococci (*p* = 0.0010) in the Probiotic group compared to the Control group. Viable numbers of staphylococci, bifidobacteria, *Bacteroides*, clostridia, total aerobes and total anaerobes were similar between groups. Numbers of *Firmicutes* were estimated by combining numbers of *Lactobacillus*, Clostridia, *Enterococcus* and *Staphylococcus* with the numbers of *Bacteroides* represent *Bacteroidetes* and the F:B was found to be lower in the Probiotic mice compared to the Control group (Figure 7B), agreeing with the NGS outcomes (Figure 5C).

## Discussion

Using a high fat diet to exacerbate pathological changes, inflammation and metabolic deficits in 3xTg-AD mice, we have demonstrated improvements in recognition memory and hippocampal spine density and a reduction in weight gain in response to supplementation with the Lab4b probiotic.

Behavioural analyses using Novel Object Recognition (NOR) revealed a significantly higher discrimination ratio (DR) in the Probiotic mice compared to Control mice indicating a beneficial impact upon recognition memory sensitivity (Sivakumaran et al., 2018). Mouse behaviour remained similar between groups during open field testing that, as proposed by Bonfili et al. (2017), verifies the outcomes of NOR testing by eliminating the possible influence of altered locomotion. The improvement in memory observed in the mice in the Probiotic group occurred alongside increased mRNA expression of several memory-related brain markers including BDNF, CPLX2, and GRIA1. BDNF is considered vital for the maintenance of neuronal function impacting upon learning and memory and reduced expression has been observed in the AD brain (Miranda et al., 2019). CPLX2 encodes a protein involved in neurotransmitter release whilst GRIA1 encodes an excitatory glutamate (AMPA) receptor and deficiencies in the expression of each have been detected in the AD brain (Ramos-Miguel et al., 2017; Williams et al., 2021). These findings align with other studies showing that dietary combinations of lactobacilli and bifidobacteria can prevent cognitive decline in ageing rats (O’Hagan et al., 2017), AD mice (Bonfili et al., 2017) and in a human AD population (Akbari et al., 2016).

The loss of recognition memory sensitivity in 3xTg-AD mice has been linked to neurodegeneration in the hippocampus (Rossato et al., 2007; Broadbent et al., 2010; Radiske et al., 2017), particularly the CA1 sub-region during early disease (Perez-Cruz et al., 2011), and significantly lower neuronal spine density was observed in the CA1 region of the hippocampus in the Control mice compared to the group receiving Probiotic indicating better neuronal health and spinal plasticity. The morphological subtyping of dendritic spines, particularly in relation to spine head size, correlates with the functional attributes of the synapse and reflects synaptic plasticity during memory and learning. Our data suggests that thin dendritic spines, but not stubby or mushroom, are amenable to preservation with probiotic supplementation. Thin spine subtypes are highly motile spines crucial for activity-dependant formation and strengthening of synapses capable of expansion during learning. The preferential loss associated with thin spines are associated with deficits in spatial learning and memory, and a feature linked with neurodegenerative diseases and normal ageing (Dumitriu et al., 2010).

In support of these findings, we observed higher transcript levels of NSMF, a regulator of the DNA repair response (Ju et al., 2021), and BDNF that is involved in neuronal survival in the whole brain extracts of the mice receiving Lab4b to the Control group. A recent study of Sun M. et al. (2021) demonstrated the ability of another probiotic to increase spine densities in the cortex of 3xTg-AD mice. Our own studies have also shown beneficial effects of probiotics on neuronal health *in vitro* with Lab4b demonstrating an ability to prevent loss of viability in human SH-SY5Y neurones exposed to neurotoxic challenge (Michael et al., 2019). *In vivo* the neurodegenerative process can result in brain shrinkage and, while no differences in brain weight were observed in the current study, other studies using a multi-strain probiotic have been shown to rescue brain weight and volume loss in 6-month old 3xTg-AD mice (Bonfili et al., 2017).

Neuro-inflammation as part of the progression of AD (Hemonnot et al., 2019) involves an influx/activation of microglial cells and macrophages in the brain (Hopperton et al., 2018) but no differences in cell numbers or microglial activation were observed in the cortical and hippocampal regions of the Probiotic mice in relation to the Control. The gene expression levels of inflammatory markers were assessed and there was less expression of IL-18, NLRP1a, NLRP1b, and Caspase-1 in the brains of Probiotic mice compared to the Control mice which we conclude shows an impairment of inflammasome activity in this group. Elevated transcript levels of the pro-inflammatory cytokines IL-18 and IL-1β have been observed in the brains of AD patients (Ojala et al., 2009; Saresella et al., 2016) and the secretion of these cytokines is modulated by inflammasomes that are caspase-1 activating multi-protein complexes involved in the activation of the innate immune response and cell death via pyroptosis (Kelley et al., 2019). It is worthy of note that, contrary to expectation, the expression of NLRC4, an inflammasome component associated with the augmentation of neuro-inflammation and cognitive decline during AD (Saadi et al., 2020), was significantly higher in the probiotic fed mice compared to the control although elevations did not adversely affect the expressions levels of IL-1β and IL-8 in the whole brain extracts of these mice.

IL-10, an anti-inflammatory cytokine associated with the resolution of neuro-inflammation (Kwilasz et al., 2015), was higher in the Probiotic group compared to the Control group. The over expression of IL-10 in the brains of AD mice has been associated with improved cognitive performance (Kiyota et al., 2012) although the role of IL-10 during AD is both complex and contentious; IL-10 deficiency has also been shown to reduce disease pathology in AD mice (Guillot-Sestier et al., 2015).

Changes in the levels of circulatory cytokines are often observed during AD (Walker et al., 2019) and metabolic disease (Guarner and Rubio-Ruiz, 2015) and higher levels of IL-10 were observed in the circulation of the Control mice compared to the Probiotic mice. As previously mentioned, IL-10 is generally considered to be anti-inflammatory and it is plausible that higher levels in the Control group form part of the host response to the overtly inflammatory systemic environment that was absent in the Probiotic mice. It has also been shown that, in some instances, elevated levels of IL-10 can hinder the resolution of inflammation and drive disease progression in murine models of AD (Chakrabarty et al., 2015) thus supporting the anti-inflammatory capability of the probiotic. A significant between-group reduction in the circulating levels of the pro-inflammatory cytokine, TNF-α and pro-inflammatory chemokine, KC/GRO, were observed at the end of the study in favour of the mice receiving Lab4b. Similar findings have been observed in healthy Wistar rats receiving a similar probiotic intervention (Webberley et al., 2021).

Obesity and high cholesterol increase the risk of development of AD (Anstey et al., 2011; Saiz-Vazquez et al., 2020) and metabolic disease states such as cardiovascular disease (Silverman et al., 2016). Weight gain and circulating levels of LDL-cholesterol in the Probiotic group were lower than those of the Control group supporting the findings of our own previous study in HFD-fed wild-type C57BL/6J mice (Michael et al., 2017) and a recent study showing the hypocholesterolaemic effects of the SLAB51 multistrain probiotic in 3xTg-AD mice (Bonfili et al., 2022). The liver is the master regulator of host metabolism [with dysfunction linked to the progression of AD (Estrada et al., 2019)] and higher hepatic transcript levels of CD36 and PPAR-γ were observed in the Control mice compared to the mice receiving probiotic. CD36 and PPAR-γ play key roles in lipid-homoeostasis and increased hepatic mRNAs have been associated with abnormal storage of fat and hepatic steatosis (Liss and Finck, 2017; Rada et al., 2020). SREBP-2 plays a key role in the biosynthesis of cholesterol in the liver by regulating the expression of the cholesterol synthesising enzyme 3-Hydroxy-3-Methylglutaryl-CoA Reductase (HMGCR; Luo et al., 2020). Lower SREBP-2 expression was observed in the Control group and the expression of HMGCR was 40% higher in the Probiotic group compared to the Control (non-significant, see Supplementary Table 5), possibly indicating the presence of a compensatory mechanism to normalise the Lab4b-mediated reduction in LDL-cholesterol levels (Ness et al., 2006). NMR analysis highlighted significantly higher glycogen and glucose in the Control group compared to the Probiotic group suggesting a potential for the probiotic to impact upon glucose and insulin tolerance. There is a growing awareness that perturbations in glucose/insulin metabolism can contribute to the development of AD, such that AD has been branded “diabetes of the brain” (Nguyen et al., 2020), and there is evidence supporting the anti-diabetic effects of probiotics (Cao et al., 2021).

Metabolic disorders and AD are associated with perturbations of the gut microbiota (Zhuang et al., 2018; Dabke et al., 2019) and 16S rRNA gene sequencing of faecal DNA identified significant reduction in the Firmicutes:Bacteroidetes (F:B) ratio in the Probiotic group. A high (F:B) is a characteristic of obesity (Stojanov et al., 2020), therefore these findings are consistent with the prevention of weight gain observed in these mice. BLAST analysis of enriched ASVs found the *Blautia* genera to be associated with the Control mice. The *Blautia* genus has been found to be associated with fat accumulation in humans and so this is consistent with the weight gain in these mice (Ozato et al., 2019). Within the Probiotic mice, *Ligilactobacillus murinus*, which has been shown to play a role in maintaining a healthy microbiota, was found to be more abundant. This suggests that the probiotic could be causing beneficial changes within the microbiome (Qi et al., 2019).

Analysing the faecal microbiota using microbiological techniques to enumerate the selected populations; probiotic supplementation revealed significant reductions in lactobacilli, enterobacteria, coliforms and yeast, while an increase in the enterococcal component of the microbiota was observed. Enterobacteria are enriched during AD (Liu et al., 2019) and have been shown to drive disease progression in *Drosophila* (Wu et al., 2017). It is emerging that commensal intestinal yeast (the gut mycobiome) can contribute to both the progression of AD (Nagpal et al., 2020) and HFD-induced obesity (Sun S. et al., 2021). however, it remains unclear and more investigation is needed. Furthermore, no significant change in the relative abundance of the *Lactobacillus* genus was observed in the Probiotic group compared to the Control (data not shown), thus, the number of viable but non-culturable bacteria could be an influence (Ayrapetyan and Oliver, 2016). Estimation of the F:B ratio from the *ex vivo* analysis of microbial numbers showed indication of a reduction in the Probiotic group compared to the Control group in alignment with the NGS data presented in Figures 5, and 6.

There are a number of limitations to our study: (i) The absence of a longitudinally chow-fed control group means we cannot confirm the detrimental effects of the HFD although this was not the key aim of the study and has been demonstrated on numerous occasions elsewhere (Julien et al., 2010; Knight et al., 2014; Kim et al., 2017). (ii) We were unable to assess the impact of Lab4b on amyloid deposition due the absence of amyloid plaques (a hallmark of AD) in the brain tissue of our animals. There is some evidence that phenotypic loss that can occur during the generation of sublines of 3xTg-AD mice (Belfiore et al., 2019) or it is possible that the absence of plaque is linked to the relatively young age of the mice. (iii) Cognitive function was assessed using only the NOR test thus limiting our understanding of the effects of Lab4b supplementation to a single cognitive domain. More comprehensive analysis was unfortunately limited in the study due to the limited space available when carrying out behavioural analyses in class II laminar flow hoods. This meant that tests such as the Morris water maze could not be applied and even the commonly used Y-maze did not fit within the flow hoods that were available. These restrictions were necessitated by the conduct of the work in a facility used for immunological studies. Such work requires housing of experimental mice in scantainers and husbandry (changing bedding, food, etc.) being carried out in class II laminar flow hoods. A battery of tests are required in future work to fully assess the cognitive effects of Lab4b. (iv) The rates of food intake were not measured so it is unclear if the probiotic-mediated weight loss is due to changes in lipid metabolism and/or satiety. (v) Only one dose of Lab4b was tested so the observed effects may not be optimal; however, the dose was selected to reflect a realistic human equivalent dose (Nair and Jacob, 2016).

In summary, this 3-month study in 3xTg-AD mice receiving a high fat diet demonstrated the ability of the Lab4b probiotic to slow cognitive decline and the progression of AD pathology in the midst of metabolic challenge while imparting metabolic improvements including the limitation of weight gain and lowering of plasma lipids. These findings support the potential use of probiotic bacteria to impair the progression of AD and highlight a holistic capability for the attenuation of a number of metabolic risk factors of AD and other metabolic disease states. On the basis of these findings there is support for translation in future human studies.

## Data Availability Statement

The data presented in the study are deposited in the European Genome-Phenome Archive (EGA) repository, accession number PRJEB51991.

## Ethics Statement

The animal study was reviewed and approved by the Animal Welfare and Ethical Review Body (AWERB), Cardiff University. Written informed consent was obtained from the owners for the participation of their animals in this study.

## Author Contributions

TH, TW, DM, and SP were responsible for the design of the study. TW, RB, JK-S, AJ, ST, MG, JL, and JRM performed the experiments. GM, DJ, TW, and DM performed the data and statistical analyses. JEM, JRM, and MAG provided assistance and knowledge that was vital to the completion of the manuscript. DM, SP, TW, and DJ prepared the manuscript. All authors contributed to the review of the manuscript.

## Conflict of Interest

TW, GM, JK-S, AJ, DM, ST, DJ, and SP were employed by the Cultech Ltd. The funder had the following involvement in the study: the design of the study. The remaining authors declare that the research was conducted in the absence of any commercial or financial relationships that could be construed as a potential conflict of interest.

## Publisher’s Note

All claims expressed in this article are solely those of the authors and do not necessarily represent those of their affiliated organizations, or those of the publisher, the editors and the reviewers. Any product that may be evaluated in this article, or claim that may be made by its manufacturer, is not guaranteed or endorsed by the publisher.
