# Supplementary material for: The Impact of Probiotic Supplementation on Cognitive, Pathological and Metabolic Markers in a Transgenic Mouse Model of Alzheimer’s Disease
Source: Front Neurosci. 2022 May 24;16:843105. doi: 10.3389/fnins.2022.843105 (PMC9172594; doi:10.3389/fnins.2022.843105)
Supplement: Supplementary file 1 [file Data_Sheet_1.docx]

**Supplementary data: The impact of probiotic supplementation on cognitive, pathological and metabolic markers in a transgenic mouse model of Alzheimer’s disease**

**NMR - Aqueous and organic extraction from tissue**

Brain tissues including cortex, hippocampus and cerebellum, and liver tissue were processed for extraction of polar and lipophilic metabolites as previously described3,4. Briefly, tissue weight was recorded, and samples were placed in ice-cold methanol (MeOH): water (H20) solution (1:1; v:v) in Zirconia bead containing tubes (BioSpec products supplied by Stratech Scientific, UK) for use in the Precellys 24 tissue homogenizer (Bertin supplied by Exensor, UK). Tissue was homogenized using three rounds of 40 s per cycle followed by cooling on dry ice and then another further beating for 40 s at 6500 Hz. After centrifugation at 10, 000 g for 10 min at 4°C, the supernatant was collected and transferred to a new Eppendorf tube. This sample constituted the aqueous extract (water-soluble metabolites) and was dried in an Eppendorf vacuum concentrator plus (Eppendorf, Stevenage, UK) for 3 h at 45°C at V-AQ (vacuum aqueous mode). Samples were stored at -40°C until further use for 1H NMR analysis. The remaining tissue pellet on the bead containing tube was kept on dry ice during the entire procedure. Pre-chilled dichloromethane (DCM): MeOH (3:1; v:v) was added to the tissue pellet for the preparation of organic extracts (lipid metabolites). Samples were bead-beaten again as in the previous step and then centrifuged at 10 000 g for 10 min at 4°C. Supernatant was collected and transferred into glass vials and was left to air-dry overnight. Samples were stored at -40°C until further use. The organic phase was not analysed herein since we were interested in the aqueous phase metabolites.

**NMR - Sample preparation for 1H NMR spectroscopic analysis of tissue, plasma and faecal extracts**

Different approaches were followed for the preparation of tissue, faecal and plasma samples before NMR processing. Faecal samples were weighted (~30-50 mg) and resuspended in 20 times diluted 1.5M potassium phosphate KH2PO4, which contained 100% D2O for magnetic field lock, 1% 3-trimethylsilyl-1-[2, 2, 3, 3-d4] sodium propionate (TSP) for spectral calibration, and 2 mM NaN3 (pH= 7.4) to avoid contamination, using D2O at a ratio 1:4 (mg of faecal weigh: µl of resuspension buffer). After three cycles of sonication (10 min, 25 °C) and vortexing (20s) to maximize the solubility of metabolites, samples were centrifuged at 18 000 g for 20 min at 4 °C. 60 µl of supernatant from each sample was taken and transferred to 1.7 mm diameter micro-NMR tube for 1H NMR analysis.

For tissues, the process differed depending on whether the samples were run in 3 mm or 1.7 mm diameter NMR tubes. For 3mm NMR tubes, polar tissue extracts (cortex and liver) were re-suspended in 220 µl D2O (the volume added was adjusted based on the weight of the sample and 220µl corresponds to the amount added to the sample with the smallest weight). Samples were dissolved by 3 rounds of sonication followed by vigorous vortexing after each round. After centrifugation at 10 000 g for 5min at 4°C, 180 µl of supernatant was taken and mixed with 20 µl of the aforementioned phosphate buffer in a new Eppendorf tube (1 in 10 dilution). All the mixtures were vortexed, spun down at 10 000 g for 5 min at 4°C and 180 µl of supernatant was transferred to 3 mm tubes for NMR analysis. For 1.7 mm NMR tubes, 80 µl of 20 times diluted phosphate buffer in D2O was added in polar tissue extracts (hippocampus and cerebellum) (the volume added was adjusted based on the weight of the sample and 80 µl corresponds to the amount added to the sample with the smallest weight). After three rounds of sonication followed by vigorous vortexing, samples were spun down at 10 000 g for 5 min at 4°C and 60 µl of supernatant was transferred to 1.7 mm NMR tube for further analysis.

For plasma, samples were defrosted and spun down at 20 000 g for 10 min at 4°C. A total of 100 µl of plasma supernatant was mixed with 100 µl of plasma buffer (75 mM Na2HPO4, 2mM NaN3, 0.08% TSP, 0.2% D2O solution, pH =7.4). The mixtures were vortexed, spun down at 10 000 g for 5 min at 4°C and 180 µl of supernatant was transferred into an NMR tube with an outer diameter of 3mm pending NMR analysis.

**1H NMR spectroscopic analysis of tissue, plasma and faecal extracts.**

1H NMR spectra of all samples were acquired using a Bruker DRX 600 MHz spectrometer (Bruker, Rheinstetten, Germany) operating at 600.13 MHz. A standard NMR pulse sequence for 1D experiment [recycle delay (RD)-90° t1-90°-tm-90°-acquire free induction decay (FID)] was employed at a constant temperature of 300 K for the acquisition of tissue, plasma and faecal spectra 1,2. Water signal of biofluids was suppressed through presaturation, while D2O solvent was used to lock the magnetic field signal. The water peak was suppressed by irradiation during the RD of 4 s and mixing time (tm) of 100 ms. A total of 32 scans were recorded into 64 k data points with a spectral width of 20 ppm for cortex and liver, whereas a total of 64 scans were acquired into 64 k data points with a spectral width of 20 ppm for cerebellum and hippocampus. For each faecal sample a total of 128 scans was collected into 65k data points with a spectral width of 20 ppm. For plasma, a total of 128 scans was accumulated into 98 k data points with a spectral width of 30 ppm. In order to highlight signals from low molecular weight metabolites, a CPMG (Carr-Purcell-Meiboom-Gill) pulse sequence [RD−90°− (τ−180°−τ)n− acquire FID] was additionally applied to all plasma samples at 310 K using a spectral width of 20 ppm and 128 scans with 64 k data points. The receiver gain was set to 90.5 for all the experiments except for plasma that it was set to 16.

**1H NMR data processing and multivariate statistical analysis.**

1H NMR spectra were Fourier transformed and automatically pre-processed in Topspin 3.3 (Bruker, Germany). Prior to Fourier transformation, an exponential function equivalent to a line broadening of 0.3 Hz was used to multiply FIDs. Pre-processing encompassed phasing, baseline correction and referencing to TSP peak at δ 0.0 apart from plasma spectra that were referenced using the anomeric proton assigned to α-glucose at δ 5.22. Phasing and baseline correction for plasma spectra were performed manually in MATLAB (Mathworks, USA) using in-house developed scripts. The resulting NMR spectra (δ 0-10ppm) were imported to MATLAB with a resolution of 0.0005 ppm. The water peak regions δ 4.7 - 4.9 for hippocampus, cerebellum and liver, δ 4.7 -4.82 for cortex, δ 4.7 -4.92 for faecal extracts, , δ 4.5 -4.9 for plasma NOESY and δ 4.6 -4.9 for plasma CPMG were removed to minimise the effect of the distorted baseline caused by imperfect water suppression. Additionally, regions δ 0 – 1.2 for hippocampus, δ 0-1 for cerebellum, δ 0-0.2 for liver, δ 0-0.8 for cortex, δ 0-0.4 for faecal extracts, δ 0-0.2 for plasma standard 1D spectra and CPMG spectra containing only noise were cut out. Remaining spectra were aligned using the recursive segment-wise peak alignment (RSPA) algorithm to correct for chemical shift variations that may introduce unwanted systematic variation compromising results and statistical analysis5. Normalization using the probabilistic quotient method 6 was performed prior to any multivariate statistical analysis. Unsupervised methods such as principal component analysis (PCA)7 and supervised methods such as orthogonal signal correction-projection to latent structures-discriminant analysis (O-PLS-DA)8 with a unit variance (UV) scaling method were applied using SIMCA version 15 (Umetrics, Sartorius Stedim Biotech) and MATLAB. The validity and robustness of the all the O-PLS-DA models was assessed using internal cross-validation (typically 7-fold) and permutation testing. The main parameters calculated by these models are R2X and Q2Y, reflecting the variation in X matrix (i.e. 1H NMR spectra) explained by the model and the predictability of the model, respectively. OPLS-DA is helpful in the identification of metabolites that contribute to the discrimination between two sample groups8. To identify significant discriminant metabolites, univariate statistical analysis was carried out using t-tests followed by Benjamini-Hochberg multiple testing correction9. P values <0.05 were considered statistically significant. Statistical Total Correlation Spectroscopy (STOCSY)10 was performed to assist in metabolite assignment along with the use of Chenomx NMR Suite (Chenomx, Canada), in-house databases and public datasets 11.

**References**

1 Beckonert, O. et al. Metabolic profiling, metabolomic and metabonomic procedures for NMR spectroscopy of urine, plasma, serum and tissue extracts. Nat Protoc 2, 2692-2703, doi:10.1038/nprot.2007.376 (2007).

2 Dona, A. C. et al. Precision high-throughput proton NMR spectroscopy of human urine, serum, and plasma for large-scale metabolic phenotyping. Anal Chem 86, 9887-9894, doi:10.1021/ac5025039 (2014).

3 Want, E. J. et al. Global metabolic profiling of animal and human tissues via UPLC-MS. Nat Protoc 8, 17-32, doi:10.1038/nprot.2012.135 (2013).

4 Vorkas, P. A. et al. Untargeted UPLC-MS profiling pipeline to expand tissue metabolome coverage: application to cardiovascular disease. Analytical chemistry 87, 4184-4193, doi:10.1021/ac503775m (2015).

5 Veselkov, K. A. et al. Recursive segment-wise peak alignment of biological (1)h NMR spectra for improved metabolic biomarker recovery. Anal Chem 81, 56-66, doi:10.1021/ac8011544 (2009).

6 Dieterle, F., Ross, A., Schlotterbeck, G. & Senn, H. Probabilistic quotient normalization as robust method to account for dilution of complex biological mixtures. Application in 1H NMR metabonomics. Anal. Chem. 78, 4281-4290, doi:10.1021/ac051632c (2006).

7 Lindon, J. C. & Nicholson, J. K. Spectroscopic and statistical techniques for information recovery in metabonomics and metabolomics. Annu Rev Anal Chem (Palo Alto Calif) 1, 45-69, doi:10.1146/annurev.anchem.1.031207.113026 (2008).

8 Beckwith-Hall, B. M. et al. Application of orthogonal signal correction to minimise the effects of physical and biological variation in high resolution 1H NMR spectra of biofluids. The Analyst 127, 1283-1288 (2002).

9 Benjamini, Y. & Hochberg, Y. Controlling the false discovery rate: a practical and powerful approach to multiple testing. J. R. Statist. Soc. 57, 289-300 (1995).

10 Cloarec, O. et al. Statistical total correlation spectroscopy: an exploratory approach for latent biomarker identification from metabolic 1H NMR data sets. Anal Chem 77, 1282-1289, doi:10.1021/ac048630x (2005).

11 Wishart, D. S. et al. HMDB 4.0: the human metabolome database for 2018. Nucleic Acids Res 46, D608-D617, doi:10.1093/nar/gkx1089 (2018).
