# Supplementary material for: The Impact of Probiotic Supplementation on Cognitive, Pathological and Metabolic Markers in a Transgenic Mouse Model of Alzheimer’s Disease
Source: Front Neurosci. 2022 May 24;16:843105. doi: 10.3389/fnins.2022.843105 (PMC9172594; doi:10.3389/fnins.2022.843105)
Supplement: Supplementary file 2 [file Table_1.docx]

**Supplementary data: The impact of probiotic supplementation on cognitive, pathological and metabolic markers in a transgenic mouse model of Alzheimer’s disease**

**
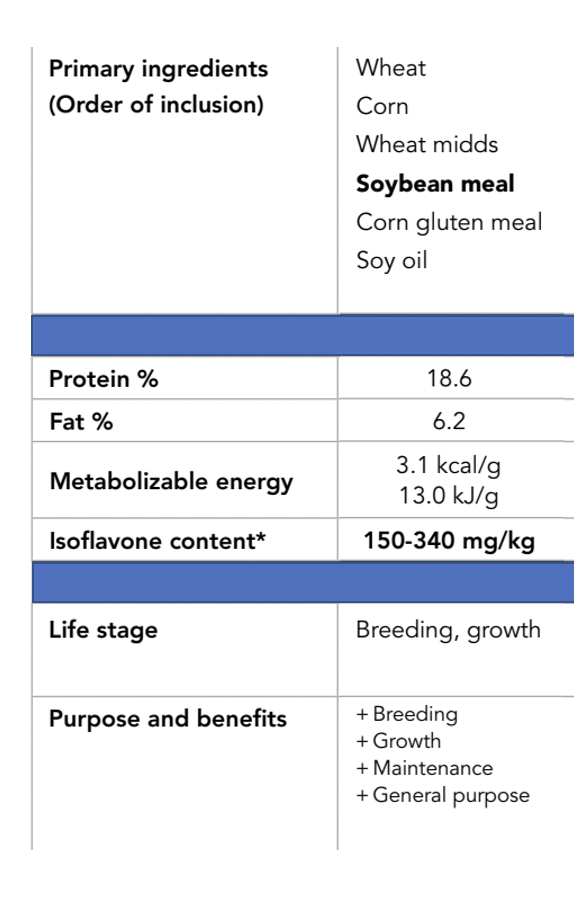
**

**Supplementary Table S1.** Compositional breakdown of the chow diet. Data are obtained and modified from Teklad diet formulations provided by Envigo.

**
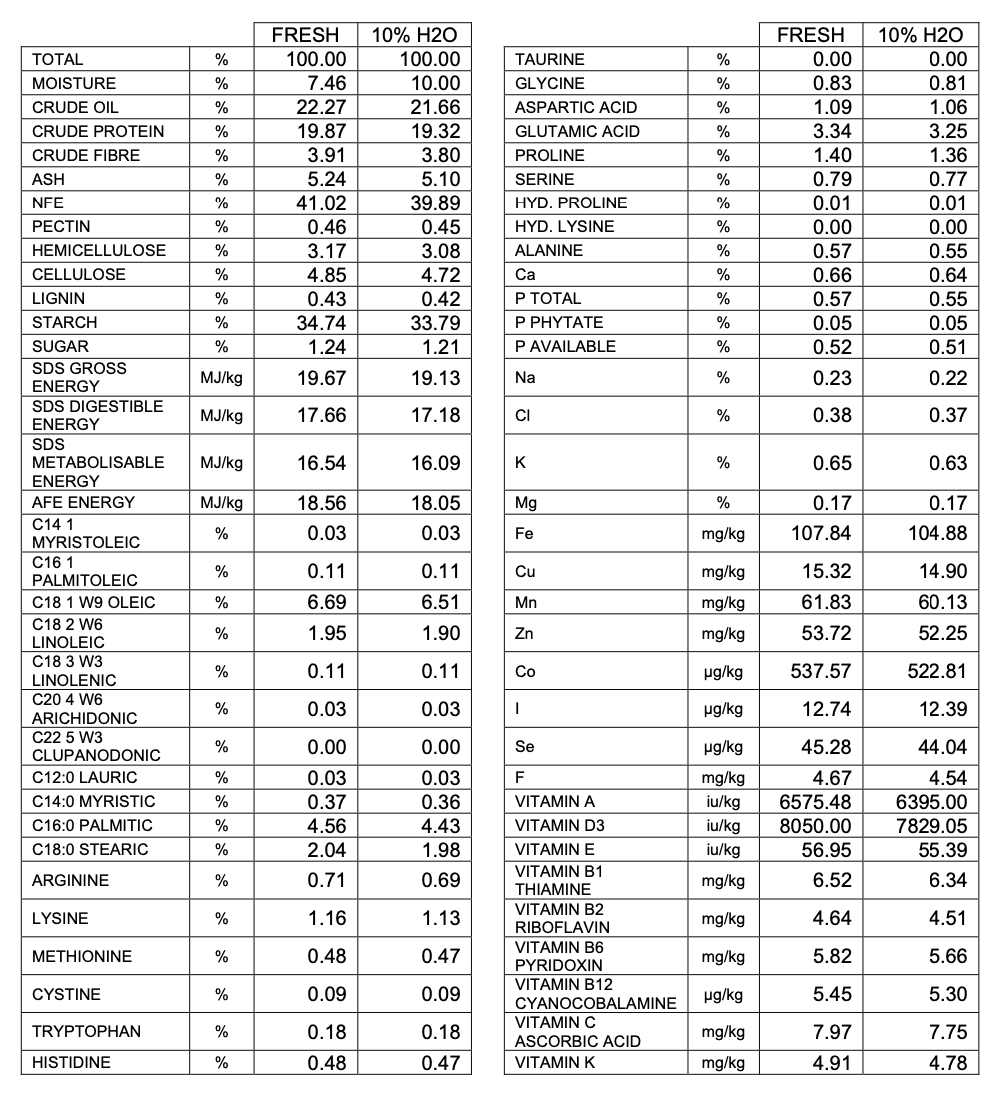
**

**
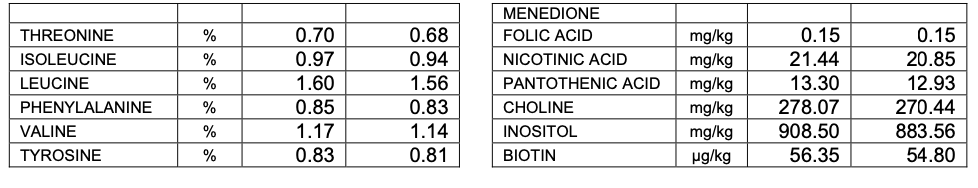
**

**Supplementary Table S2.** Compositional breakdown of the HFD. Data tables obtained and modified from Special Diet Services (SDS).

| Tissue | **Gene Function** | **Gene** | **Forward primer (5’-3’)** | **Reverse Primer (5’-3’)** |
| --- | --- | --- | --- | --- |
| Brain | **Cognition/Neuronal function** | BDNF | GGGTCACAGCGGCAGATAAA | GCCTTTGGATACCGGGACTT |
|  |  | CPLX-2 | AGTGGCTTAGACGGTTGCTG | TGGTGGCCCCTCCGA |
|  |  | GRIA1 | TGGTGGCCCCTCCGA | GCTCAGAGCACTGGTCTTGT |
|  |  | NSMF | TGATGTCCCCATCCGTACCT | CCCCTGAATACCGAGATGGC |
|  |  | CPLX1 | GACCCCGATGCTGCTAAGAA | TGCCATACTTGTCTCTTATACCCT |
|  |  | GRIN1 | GTCCAGCGTCTGGTTTGAGA | TTCTCTGCCTTGGACTCACG |
|  |  | GPR41 | TCCAATACTCTGCATCTGTGACC | CATCACGTTGAGGGGGAGTC |
|  |  | GPR109A | CTTCCTACCCAGTGTGGCTG | CAGGTCCACCGAGGAGTAGA |
|  |  | APP | GACCACTCGACCAGGTTCTG | ACACCAGTTTTTGATGGCGG |
|  |  | PSEN-1 | TATACCCGGAAGGACGGTCA | CAGGCGTGGATGACCTTGTA |
|  | **Apoptosis** | BAX | TGCAGAGGATGATTGCTGAC | GATCAGCTCGGGCACTTTAG |
|  |  | BCL2 | TGAGTACCTGAACCGGCATCT | GCATCCCAGCCTCCGTTAT |
|  | **Inflammation/Inflammasome** | IL-10 | TAAGGGTTACTTGGGTTGCCA | GAGAAATCGATGACAGCGCC |
|  |  | IL-18 | CAAGTTTACAAGCATCCAGGCA | CATTGTTCCTGGGCCAAGAGG |
|  |  | NLRP1b | TCCAGTGGAAGAGTGGGGTCT | GGAGCTCTCGGATACTGCTTC |
|  |  | NLCR4 | ACAGTTGTCTCTCATGGACAAAGA | CTTGGCAAGGCTCTCCTGG |
|  |  | Caspase-1 | CTATGGACAAGGCACGGGAC | TCAGCTGATGGAGCTGATTGA |
|  |  | IL-6 | GAGGATACCACTCCCAACAGACC | AAGTGCATCATCGTTGTTCATACA |
|  |  | IL-5 | AGCAATGAGACGATGAGGCTT | TCCTCGCCACACTTCTCTTTT |
|  |  | IL-1B | TGCCACCTTTTGACAGTGATG | TGATGTGCTGCTGCGAGATT |
|  |  | NLRP3 | GACACGAGTCCTGGTGACTT | GGCTTAGGTCCACACAGAAAG |
|  |  | NLRP1a | CATAGAGGAGCAGGCAGGTCTC | TGCTTCAAGACGCTCTTCTGT |
|  |  | KC-GRO | ACCGAAGTCATAGCCACACTC | ACTTGGGGACACCTTTTAGCA |
|  |  | AIM2 | GGTGGCGTCAGGAAGTTTTC | GCCGGTCAACAACAGCATTT |
| **Liver** | **Lipid homeostasis** | SREBP-2 | GGCACACTGCAGACCCTTGC | GAAGGGCGGCTGTCTGGATG |
|  |  | CD36 | GAATGGGCTGTGATCGGAAC | ACGTCATCTGGGTTTTGCAC |
|  |  | PPAR-γ | TGTGGGGATAAAGCATCAGGC | CCGGCAGTTAAGATCACACCTAT |
|  |  | FXR | TGAGAACCCACAGCATTTCG | GCGTGGTGATGGTTGAATGTC |
|  |  | CYP7A1 | GCGTGGTGATGGTTGAATGTC | TCACTTCTTCAGAGGCTGGTTTC |
|  |  | CYP8B1 | GAACTCAACCAGGCCATGCT | AGGAGCTGGCACCTAGACT |
|  |  | SHP-1 | TCTGCAGGTCGTCCGACTATTC | AGGCAGTGGCTGTGAGATGC |
|  |  | HMGCR | AAGGGTACGGAGAAAGCACT | AATGACGCTTCACAAACCA |
|  | **Oxidative defence** | SOD1 | CCAGTGCAGGACCTCATTTT | CACCTTTGCCCAAGTCATCT |
|  |  | SOD2 | GGCCAAGGGAGATGTTACAAC | GCAACTCTCCTTTGGGTTCTC |
|  |  | SOD3 | TTCTTGTTCTACGGCTTGCTAC | CTCCATCCAGATCTCCAGCACT |
|  |  | GPX1 | CAGGAGAATGGCAAGAATGAAGAG | GGCATTCCGCAGGAAGGTAAAGAGCGG |
|  |  | CAT | GCGGATTCCTGAGAGAGTGGTAC | GCCTGACTCTCCAGCGACTGTGGAG |
|  |  | GSR | CCAATGTCAAAGGCGTCTATG | AGACCACAGTAGGGATGTTGTCA |

**Supplementary Table S3:** qPCR primers used in study

| Material | **ATCC #** | **Concentration (ng/μL)** | **Weight (g)** | **Genomic copy number/μL (1x)** | **Genomic Copy Number** | **RSD** |
| --- | --- | --- | --- | --- | --- | --- |
| *Staphylococcus aureus* (MRSA) | BAA-1556D-5 | 4.00E-03 | 0.80% | 1270.00 | 0.75% | 4.00% |
| *Staphylococcus aureus* (MSSA) | BAA-1718D-5 | 6.00E-02 | 12.00% | 19500.00 | 11.52% | 5.09% |
| *Streptococcus pneumoniae* | 700669 | 1.28E-01 | 25.60% | 52600.00 | 31.09% | 2.19% |
| *Streptococcus pyogenes* | 700294 | 2.00E-02 | 4.00% | 9850.00 | 5.83% | 3.49% |
| *Streptococcus galactiae* | BAA-611 | 7.50E-03 | 1.50% | 3170.00 | 1.87% | 23.84% |
| *Entercoccus faecalis* | 700802 | 5.00E-03 | 1.00% | 1370.00 | 0.81% | 3.66% |
| *Pseudomonas aeruginosa* | 47085 | 5.00E-03 | 1.00% | 728.00 | 0.43% | 9.17% |
| *Klebsiella pneumoniae* | 700721 | 1.20E-01 | 24.00% | 20600.00 | 12.18% | 4.34% |
| *Acinetobacter baumannii* | 17978 | 5.00E-04 | 0.10% | 114.00 | 0.07% | 20.76% |
| *Escherichia coli* | 700928 | 1.00E-02 | 2.00% | 1740.00 | 1.03% | 20.43% |
| *Neisseria meningitidis* | 700532 | 1.40E-01 | 28.00% | 58200.00 | 34.42% | 13.65% |
| TOTAL |  | 5.00E-01 | 100.00% | 1.69E+05 | 100.00% |  |

**Supplementary Table S4:** MCM1 composition

| Tissue | **Gene Function** | **Gene** | **Control** | **Probiotic** |
| --- | --- | --- | --- | --- |
| Brain | **Cognition/Neuronal function** | CPLX1 | 1.00±0.120 | 1.129±0.158 |
|  |  | GRIN1 | 1.00±0.072 | 1.114±0.028 |
|  |  | GPR41 | 1.00±0.380 | 1.034±0.270 |
|  |  | GPR109A | 1.00±0.264 | 0.359±0.122 |
|  |  | APP | 1.00±0.085 | 1.133±0.058 |
|  |  | PSEN-1 | 1.00±0.083 | 1.177±0.059 |
|  | **Oxidative defence** | SOD1 | 1.00±0.124 | 1.136±0.091 |
|  |  | SOD2 | 1.00±0.088 | 1.161±0.077 |
|  |  | SOD3 | 1.00±0.083 | 1.153±0.146 |
|  |  | GPX1 | 1.00±0.093 | 1.056±0.129 |
|  |  | CAT | 1.00±0.112 | 1.139±0.121 |
|  |  | GSR | 1.00±0.096 | 0.961±0.081 |
|  | **Apoptosis** | BAX | 1.00±0.105 | 0.978±0.095 |
|  |  | BCL2 | 1.00±0.090 | 1.080±0.095 |
|  |  | BAX/BCL2 | 1.00±0.092 | 0.902±0.062 |
|  | **Inflammation/Inflammasome** | IL-6 | 1.00±0.165 | 1.375±0.342 |
|  |  | IL-5 | 1.00±0.181 | 0.681±0.154 |
|  |  | IL-1B | 1.00±0.148 | 0.925±0.276 |
|  |  | NLRP3 | 1.00±0.100 | 0.848±0.170 |
|  |  | NLRP1a | 1.00±0.117 | 0.442±0.187***** |
|  |  | KC-GRO | 1.00±0.173 | 1.362±0.728 |
|  |  | AIM2 | 1.00±0.163 | 0.996±0.189 |
|  |  | CD36 | 1.00±0.124 | 0.773±0.221 |
|  | **Lipid homeostasis** | FXR | 1.00±0.269 | 0.602±0.207 |
|  |  | CYP7A1 | 1.00±0.192 | 1.160±0.392 |
|  |  | CYP8B1 | 1.00±0.290 | 0.666±0.170 |
| **Liver** | **Lipid homeostasis** | FXR | 1.00±0.122 | 0.875±0.091 |
|  |  | CYP7A1 | 1.00±0.376 | 0.879±0.223 |
|  |  | CYP8B1 | 1.00±0.107 | 1.193±0.191 |
|  |  | SHP-1 | 1.00±0.263 | 1.037±0.257 |
|  |  | HMGCR | 1.00±0.139 | 1.639±0.336 |
|  | **Oxidative defence** | SOD1 | 1.00±0.073 | 1.072±0.128 |
|  |  | SOD2 | 1.00±0.077 | 0.963±0.115 |
|  |  | SOD3 | 1.00±0.077 | 0.842±0.077 |
|  |  | GPX1 | 1.00±0.076 | 1.184±0.141 |
|  |  | CAT | 1.00±0.069 | 1.052±0.139 |
|  |  | GSR | 1.00±0.087 | 1.087±0.136 |

**Supplementary Table S5:** Gene expression levels in the liver and brains of Control and Lab4b mice (expressed in fold-change from the Control group). Data are expressed as mean ± standard error of the mean (SEM) of at least 5 mice per group where *P<0.05, **P<0.01 or ***P<0.001 versus baseline. Abbreviations: CPLX1, complexin-1; GRIN1, Glutamate receptor subunit zeta-1; APP, Amyloid precursor protein; PSEN-1, presenilin 1; SOD, superoxide dismutase; GPX1, Glutathione peroxidase 1; CAT, catalase; GSR, glutathione-disulphide reductase; BAX, BCL2 associated X apoptosis regulator; BCL2, B-cell lymphoma 2 gene; IL, interleukin; NLRP, NLR family pyrin domain; KC-GRO, keratinocyte chemoattractant/growth regulated oncogene; AIM2, Interferon-inducible protein; CD36, cluster of differentiation 36; FXR, farnesoid X receptor; CYP7A, cholesterol 7alpha-hydroxylase; CYP8B1, sterol 12-alpha-hydroxylase; SHP-1, SRC homology 2 domain-containing protein tyrosine phosphatase 1; HMCGR, 3-Hydroxy-3-Methylglutaryl-CoA Reductase.


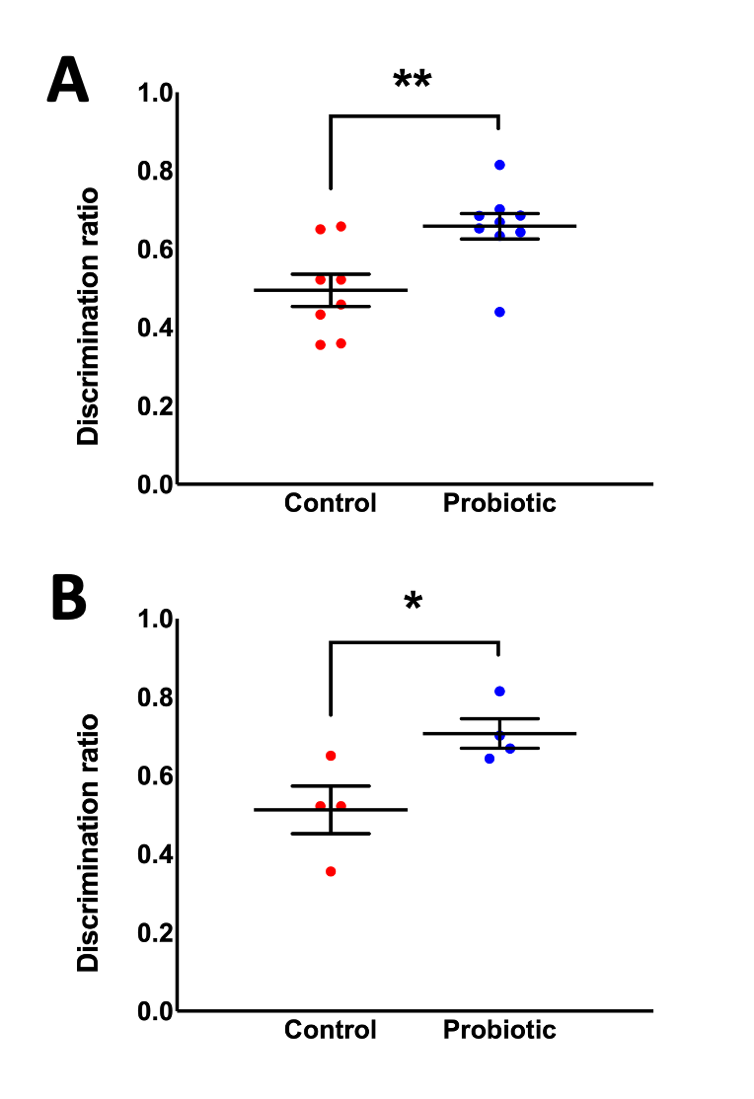


**Supplementary Figure S1**. Novel Object Recognition (NOR) test sub-grouping. Groups of 10 mice were sub-grouped according to total object exploration, in seconds, over the 10 minute NOR testing period. (A) Mice showing a total object interest time of 10 seconds of more. (B) Mice showing a total object interest of 20 seconds or more. Data are expressed as mean ± SEM. Values of p were determined using unpaired t-test where *p<0.05 and **p<0.01.

**Hippocampus**

**Cortex**

**Cerebellum**


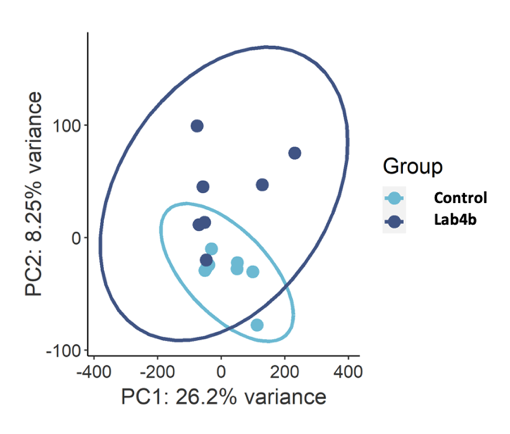

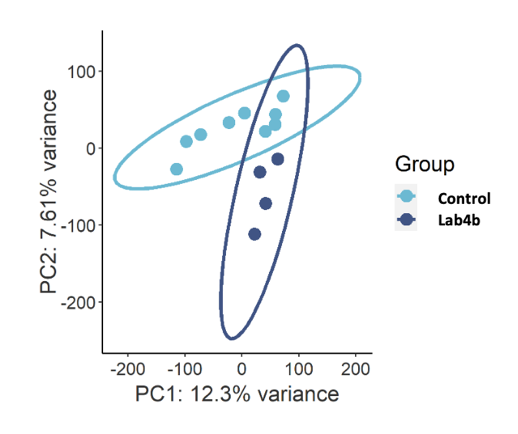

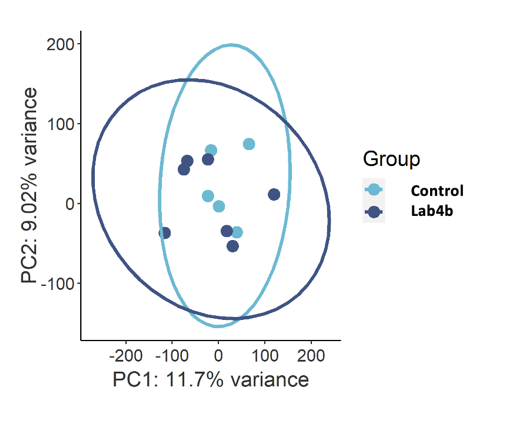


**Faeces**

**Plasma**

**
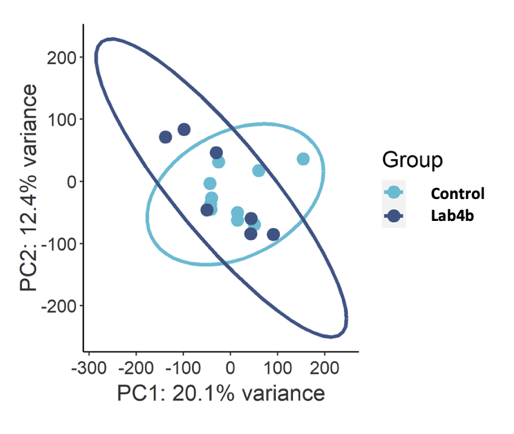

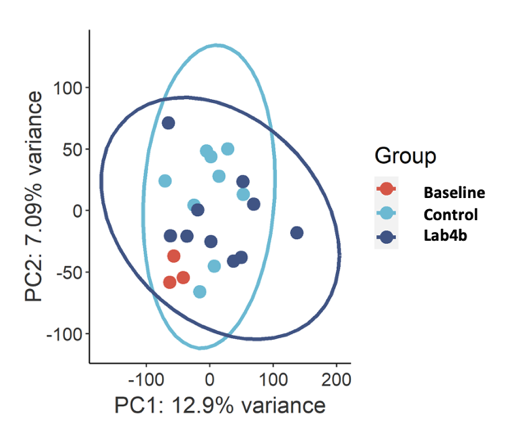
**

**Supplementary Figure S2:** Principle component analysis (PCA) of metabolites in the cerebellum, cortex, hippocampus, faeces and blood plasma of 3xTg-AD mice. Data analysis was performed on at least 8 mice per group.
